# Getting HIV Pre-exposure Prophylaxis (PrEP) into Private Pharmacies: Global Delivery Models and Research Directions

**DOI:** 10.1007/s11904-024-00696-y

**Published:** 2024-03-22

**Authors:** Stephanie D. Roche, Daniel Were, Natalie D. Crawford, Angela Tembo, Jillian Pintye, Elizabeth Bukusi, Kenneth Ngure, Katrina F. Ortblad

**Affiliations:** 1 Public Health Sciences Division, Fred Hutchinson Cancer Center, 1100 Fairview Ave N, Seattle, WA 98109 USA; 2 Jhpiego, Nairobi, Kenya; 3 Behavioral, Social, and Health Education Sciences, Rollins School of Public Health, Emory University, Atlanta, GA USA; 4 Ezintsha, Faculty of Health Sciences, University of the Witwatersrand, Johannesburg, South Africa; 5 School of Nursing, University of Washington, Seattle, USA; 6 Department of Global Health, University of Washington, Seattle, USA; 7 Department of Obstetrics and Gynecology, University of Washington, Seattle, USA; 8 Centre for Microbiology Research, Kenya Medical Research Institute, Nairobi, Kenya; 9 School of Public Health, Jomo Kenyatta University of Agriculture and Technology, Nairobi, Kenya

**Keywords:** Private pharmacies, Pre-exposure prophylaxis (PrEP), HIV prevention, Differentiated service delivery (DSD)

## Abstract

**Purpose of Review:**

To provide an overview of the current state of HIV pre-exposure prophylaxis (PrEP) delivery via private sector pharmacies globally, to discuss the context-specific factors that have influenced the design and implementation of different pharmacy-based PrEP delivery models in three example settings, and to identify future research directions.

**Recent Findings:**

Multiple high- and low-income countries are implementing or pilot testing PrEP delivery via private pharmacies using a variety of delivery models, tailored to the context. Current evidence indicates that pharmacy-based PrEP services are in demand and generally acceptable to clients and pharmacy providers. Additionally, the evidence suggests that with proper training and oversight, pharmacy providers are capable of safely initiating and managing clients on PrEP. The delivery of PrEP services at private pharmacies also achieves similar levels of PrEP initiation and continuation as traditional health clinics, but additionally reach individuals underserved by such clinics (e.g., young men; minorities), making pharmacies well-positioned to increase overall PrEP coverage. Implementation of pharmacy-based PrEP services will look different in each context and depend not only on the state of the private pharmacy sector, but also on the extent to which key needs related to governance, financing, and regulation are addressed.

**Summary:**

Private pharmacies are a promising delivery channel for PrEP in diverse settings. Countries with robust private pharmacy sectors and populations at HIV risk should focus on aligning key areas related to governance, financing, and regulation that have proven critical to pharmacy-based PrEP delivery while pursuing an ambitious research agenda to generate information for decision-making. Additionally, the nascency of pharmacy-based PrEP delivery in both high- and low-and-middle-income settings presents a prime opportunity for shared learning and innovation.

## Introduction

Successful scale-up of HIV antiretroviral therapy (ART) has required simplifying access criteria and diversifying delivery channels to meet client needs and reduce strain on healthcare systems; a similar approach will likely be needed to scale up novel HIV prevention services, including pre-exposure prophylaxis (PrEP) [1–5]. With an estimated 1.3 million people newly infected with HIV in 2022 [6] and cumulative oral PrEP initiations at 4.3 million [7], the world is not on track to meet the UNAIDS 2025 targets of new HIV infections reduced to 370,000 and “PrEP available to 10 million people at substantial risk of HIV [8](p.4).”

There is a growing consensus that private-sector pharmacies are an underutilized resource for HIV service delivery [9], including among global HIV policymakers and donors—like the World Health Organization, Global Fund, and United States (US) President’s Emergency Plan for AIDS Relief—who have called for leveraging the private pharmacy sector as one of several ways to increase access to HIV prevention services and help countries financially sustain their HIV response [3, 10, 11]. Several high- and low-and-middle-income countries, such as the USA, South Africa, and Kenya, have also identified private pharmacies as target delivery points in their national HIV/AIDS strategies [5, 12, 13].

Compared to public sector clinics, private pharmacies are typically more numerous, closer to where people live and work, and have longer operating hours. They are also a common first stop for sexual and reproductive health (SRH) products and services, such as condoms, emergency contraception, pregnancy testing, and treatment of sexually transmitted infections [14–16], making them a logical place to reach individuals who may benefit from HIV PrEP or post-exposure prophylaxis (PEP) services. However, regulation of private pharmacies varies considerably across countries, with the prevalence of illegal pharmacies low in countries with strictly enforced regulations and routine inspections (e.g., the USA and South Africa), moderate in countries that allocate fewer resources to routine enforcement but conduct periodic crackdowns (e.g., Kenya), and high in countries with both weak regulation and enforcement (e.g., Uganda).

In places with robust, organized private pharmacy sectors, the rationale for pharmacy-based PrEP delivery is sound; however, our understanding of how to operationalize it in different pharmacy settings, and the context-specific factors that may influence its feasibility, acceptability, and sustainability, are still in their early stages. This review focuses on three countries, each with > 300,000 cumulative PrEP initiations [17] that are leading in the space of private pharmacy-based PrEP delivery: the USA, Kenya, and South Africa. We will discuss how local context has influenced the design of different pharmacy PrEP models, describe early findings from programs and research studies, and identify future directions for research and implementation.

## Pharmacy PrEP Models in Use

In select parts of the USA, South Africa, and Kenya, three main pharmacy PrEP models are being implemented programmatically and/or tested via research studies: (1) a co-located model (with no task shifting), (2) a partially task-shifted model, and (3) a fully-task shifted (i.e., standalone) model, each described in Table 1. The following case studies provide specific examples of each model, with key statistics about each country’s HIV epidemic presented in Table 2.

**Table 1:** Pharmacy PrEP delivery models that have been tested or implemented in the USA, Kenya, and/or South Africa

Model	Description	Examples
**Co-located model** (i.e., no task shifting)	A cadre of healthcare worker legally authorized to initiate and manage clients on PrEP independently or under remote physician supervision (e.g., a nurse/nurse practitioner) is based in a private sector pharmacy and provides PrEP services. If involved, pharmacy providers dispense against PrEP prescriptions issued	USA: CVS Minute Clinics [18]; Walgreens HIV-specialized Pharmacies [19]; Walmart Specialty Pharmacies of the Community [20] Kenya: AGYW Nurse-Navigator Model [21••] South Africa: Pharmacies with physicians and/or NIMART-trained nurses on staff
**Partially task-shifted model**	Pharmacy providers are upskilled to deliver select components of PrEP services to eligible clients (e.g., HIV risk screening, HIV testing), with components that are beyond their scope of practice (e.g., PrEP prescribing) provided remotely via telehealth consultation by a higher-cadre healthcare worker	Kenya: Pharm PrEP Refill Model [22••] South Africa: EPIC Model [23•] prior to August 2023
**Fully task-shifted model** (i.e., standalone model)	Pharmacy providers are upskilled to independently initiate (i.e., screen, prescribe) and manage eligible clients on PrEP, with access to an HIV specialist, as needed, for consultations and referrals	USA: Kelley-Ross Pharmacy One-Step PrEP program [24] Kenya: Pharm PrEP Initiation Model [25••] South Africa: EPIC Model post-August 2023

*PrEP* pre-exposure prophylaxis, *AGYW* adolescent girls and young women, *NIMART* nurse-initiated management of antiretroviral therapy, *EPIC* Expanding ART/PrEP Innovation Consortium

**Table 2:** Key statistics about the HIV epidemics in the USA, South Africa, and Kenya

	USA	South Africa	Kenya
Population [26]	333.2 million	59.9 million	54.0 million
People living with HIV [27, 28]	1.2 million	7.5 million	1.4 million
New HIV infections in 2021 [27, 28]	36,000	210,000	35,000
HIV incidence per 1000 population [27, 28]	0.13	4.2	0.73
Total oral PrEP initiations^a^ [17]	381,784	888,217	321,662
Received oral PrEP at least once during 2021^b^ [17, 27]	227,047	346,667	117,174
Total number of private pharmacies	60,000	4700	6500

^a^Total number of individuals ever initiated on PrEP, including PrEP re-initiations following pauses in PrEP use

^b^Total number of individuals who were dispensed PrEP medication at least once during the 2021 calendar year

### Case Study 1: The United States

Across the USA, only 25% of the 1.2 million individuals who could benefit from PrEP have been prescribed it [29], with uptake particularly low among populations with disproportionate HIV burden, such as Black and LatinX individuals [30]. Key barriers to PrEP use include distance to PrEP clinics, provider bias, client distrust of the healthcare system, and cost [30]. The current US National HIV/AIDS Strategy identifies expanding the diversity of PrEP providers as a key goal, with pharmacy providers specifically called out as a workforce to engage [5]. Nationwide, there are ~ 60,000 private pharmacies, two-thirds of which are part of retail chains [31]. In July 2021, the US government clarified that most health insurance plans are required to cover the cost of PrEP medication and ancillary services [32, 33]; however, reports of private insurer non-compliance (e.g., denying claims for PrEP-related HIV testing) are not uncommon [34]. Individuals without health insurance may qualify for assistance programs—sponsored by the manufacturer [35], state [36], or federal government [37]—that cover some or all costs. For most, purchasing PrEP out of pocket is cost-prohibitive, with brand-name oral PrEP running upwards of $1800 US Dollar (USD) per month [38] and generic formulation costing ~ $60 USD per month [34].

#### Co-located Model

In recent years, large retail pharmacy chains, such as CVS, Walgreens, and Walmart, have transformed thousands of outlets into specialized pharmacies [18–20, 39, 40]. Staffed with higher-level cadres with prescribing privileges (like nurse practitioners) who have undergone HIV-specific training, these specialized pharmacies offer an array of primary care services, including HIV testing and PrEP [18–20]. If the client has public or private health insurance, the pharmacy bills the insurer directly; uninsured clients not enrolled in any assistance programs pay out of pocket. A major limitation, however, is that chain pharmacies operating this co-located model are less common in the neighborhoods with the highest need (ones with high baseline HIV incidence and poverty); the community (independently-owned) pharmacies that prevail in these neighborhoods often lack the revenue needed to sustain an in-house nurse practitioner.

#### Fully Task-Shifted Model

Select US states have also implemented the standalone model. As of July 2023, the pharmacy boards of 15 states had authorized pharmacists to prescribe PrEP under certain conditions (e.g., under a collaborative practice agreement; limited to an initial 60-day prescription [41]); enabling legislation is pending in 11 other states [42]. Currently, there is no standardized pharmacy provider PrEP delivery training, though the Pharmacy Taskforce of the Ending the HIV Epidemic in the US initiative is developing a continuing medical education program for this, and some pharmacy schools have begun incorporating PrEP delivery into the core curricula of their degree programs [43].

One example of the standalone model is the Kelley-Ross One-Step PrEP program [24] in Seattle, Washington. This program is based on collaborative practice agreements that allow pharmacists to perform certain functions under specified circumstances beyond their typical scope of practice with appropriate training and oversight by a certified drug prescriber. Since 2015, pharmacists at Kelley-Ross Pharmacy have initiated and managed > 1000 clients on oral PrEP under this model, and in 2022, they also began administering long-acting injectable PrEP [44].

A threat to the long-term feasibility of this model, however, is pharmacists’ lack of provider status at the federal level, which precludes pharmacists from being able to bill the government for clinical services rendered, even ones that fall within their scope of work (e.g., counseling, HIV testing) [45]. Since private insurers tend to follow the federal government’s payment policies [46], this limitation jeopardizes this model’s economic viability.

### Case Study 2: South Africa

Community-based, decentralized delivery of ART refills in South Africa has helped ART clients mitigate many of the same challenges currently faced by prospective PrEP clients, such as distance to clinics, long queues, and inconvenient operating hours [47]. With ~ 4700 registered private pharmacies nationwide (many with a nurse on staff at least part time) [48], the potential for private pharmacies to directly support HIV service delivery is considerable [49] and known to South Africa’s National Department of Health (NDoH). Since 2014, the NDoH has partnered with hundreds of private pharmacies across the country to serve as free ART refill collection points for stable ART clients as part of the Central Chronic Medicines Dispensing and Distribution program; however, this program does not currently include PrEP [50].

As the NDoH continues to discuss the details of its National Health Insurance scheme, there is growing pressure to establish payment and health information systems that would enable private-sector providers to deliver health services. To this end, the NDoH conducted a small pilot from 2018 to 2022 in two districts to assess the feasibility of contracting private pharmacies and physicians to deliver a predefined basket of services—including ART and PrEP—using government stock of commodities [48].

#### Co-located model

Nurses who have completed the NDoH’s course in nurse-initiated management of ART (NIMART) are permitted to prescribe ARVs in private pharmacies with appropriate permits, with physician oversight and remote consultation via a referral app. As of August 2021, 465 clients had initiated ART, PrEP, or PEP under this model, which serves both clients with private health insurance that covers PrEP and clients willing to pay out of pocket for PrEP (with name-brand and generic PrEP costing ~ $30 USD and ~ $13 USD per month, respectively [51]). In this model, uninsured clients pay separately for required laboratory exams (e.g., ~ $8 USD for hepatitis B testing). In sum, these costs are unaffordable for the 63% of South Africa’s population living below the poverty line of $6.85 USD per day [52].

#### Partially Task-Shifted Model

From August 2019 to December 2020, the Expanding ART/PrEP Innovation Consortium (EPIC)—a program of the South African HIV Clinicians Society—pilot tested a partially task-shifted model of pharmacy-based PrEP delivery in 488 private pharmacies across nine provinces [23•]. Pharmacy providers were trained to assess clients for PrEP eligibility. Once preliminary eligibility was confirmed, clients paid out of pocket to get required laboratory tests at nearby affiliated pathology labs. Upon returning to pharmacy, PrEP prescriptions were issued by a physician via telehealth consultation. Pharmacies obtained generic PrEP through their normal supply channels (i.e., no government or donor subsidies). In total, participants typically paid ~ $50 USD per month for PrEP services—an amount that pilot stakeholders viewed as a barrier to maximizing model impact [23•].

#### Fully Task-Shifted Model

Concurrent with this pilot, EPIC developed a short course similar to NIMART called pharmacist-initiated management of ART (PIMART) for training pharmacists to prescribe and manage clients on PrEP, PEP, and first-line ART [53]. Although the SAPC published enabling legislation for PIMART in August 2021 [54], a legal challenge brought forth by a group of private physicians halted implementation in December 2022 [55]. The opposing physicians argued that PIMART encroaches on the domain of medical practitioners and would compromise care quality [56]. In August 2023, the court ruled in SAPC’s favor, thereby opening up the possibility for PIMART-trained pharmacists to execute a fully task-shifted (i.e., standalone) model for pharmacy-based PrEP delivery. What clients would pay out of pocket to obtain PrEP services via this model has yet to be determined and could vary depending on factors such as private health insurance coverage and government subsidies to support this model (e.g., donated PrEP drugs).

### Case Study 3: Kenya

In Kenya, PrEP is still delivered predominantly through public channels [48]. However, in recent years, the Kenya Ministry of Health (MOH) has made private sector engagement a priority [57] and included private pharmacies as one of several target PrEP delivery points in its national framework for PrEP scale-up [13]. Across Kenya, there are ~ 6500 registered private pharmacies, most of which are owned and operated by licensed pharmaceutical technologists [48]. Starting in 2020, the MOH began granting special permissions to test pharmacy-based PrEP delivery models via research.

#### Co-located Model

From October 2020 to March 2021, a pilot study at three private pharmacies in Western Kenya tested a co-located model whereby PrEP-trained nurses (who are legally allowed to initiate clients on PrEP under remote physician supervision) were stationed at the pharmacies to offer PrEP to adolescent girls and young women (AGYW) purchasing contraception [21••]. The nurses delivered counseling, assessed HIV risk behaviors, completed HIV testing, and dispensed PrEP to all eligible AGYW. Participants received PrEP services for free, with the study covering the cost of the HIV testing kits, PrEP drug, and nurses’ time. An ongoing cluster-randomized controlled trial (cRCT; NCT05467306), launched in May 2023, is testing the effect of this co-located model compared to a standalone model (described below) on PrEP initiation and continuation outcomes among AGYW, with services available at no cost to clients [58].

#### Partially and Fully Task-Shifted Models

From November 2020 to December 2021, partially and fully task-shifted models were simultaneously pilot tested in five private pharmacies in Central and Western Kenya. In both models, trained pharmacy providers were given special privileges to deliver PrEP services using a prescribing checklist with remote physician oversight [59]. The checklist helped pharmacy providers determine clients’ eligibility to initiate PrEP (fully task-shifted model only) or continue PrEP (both models) and prompted providers to refer clients to clinic-based services if they tested HIV-positive or reported medical conditions that might contraindicate PrEP safety (e.g., history of kidney disease). At the time, the legality of pharmacy providers performing HIV rapid diagnostic testing was in dispute, so the Kenya MOH allowed the study to use provider-assisted HIVST as a workaround [59]. If clients met all checklist criteria, the pharmacy provider dispensed an initial or refill supply of PrEP (depending on the model) for a client fee of ~ $3 USD. The PrEP drug came from national stocks, with study pharmacies procuring it from nearby public clinics and submitting routine commodity reports in return. In both models, a remote physician was available for consultations and referrals.

In the partially task-shifted model, which was piloted at two public clinics, clients were initiated on PrEP by physicians at the clinics and given the option to refill PrEP at study pharmacies. In the fully task-shifted model, trained pharmacy providers provided same-day PrEP initiation and refills. Instead of undergoing creatinine clearance and hepatitis B and C testing, clients were screened for preexisting conditions that could contraindicate PrEP safety—a demedicalized approach to PrEP initiation supported by evidence from several PrEP studies which demonstrated that such testing leads to few exclusions [60, 61].

The fully task-shifted model was further tested in a 6-month extension that additionally offered PEP and removed the client fee [62••]. An ongoing cRCT (NCT05842122), launched in June 2023, is testing three variations of this standalone model compared to pharmacy referral to clinic-based PrEP services [63]. In addition to quantifying the effect of pharmacy-based PrEP delivery on PrEP initiation and continuation compared to referral, this study will also assess the effect of a client fee (free vs. ~ $2.50 USD) and pharmacy staffing (presence/absence of an HIV testing services counselor to assist with select components of PrEP delivery).

## Key Findings to Date

Table 3 summarizes key outcomes related to PrEP uptake, continuation, and acceptability of pharmacy-based PrEP delivery from the above-described examples. Additional examples and studies that are beyond the scope of this commentary are detailed in four recently published reviews [64•, 65•, 66•, 67•]. Below, we summarize five key takeaways.

1. **The demand for PrEP in pharmacies already exists, especially at pharmacies located near HIV hotspots, such as bars/nightclubs, transactional sex venues, and universities**. None of the models detailed in Table 3 featured mass media or demand creation campaigns; yet, word spread, and a considerable number of clients sought PrEP at the pharmacy and agreed to undergo PrEP screening. Notably, in two studies that charged clients a fee for PrEP services [23•, 25••], participants were willing to pay some amount, despite these same services being available free in the public sector.
2. **Pharmacies reach individuals who could benefit from PrEP, including those underserved by traditional health clinics, making them well-positioned to increase overall PrEP coverage**. In the handful of studies that report on number of clients screened for PrEP eligibility, few clients (< 15%) were found ineligible (e.g., due to testing HIV-positive) [25••, 62••, 68••]. This high eligibility rate may be attributable, in part, to pharmacies being a common resort for SRH products and services, with some clients possibly more willing to report behaviors associated with HIV risk when—by virtue of their purchases—the pharmacy provider already has some awareness of their sexual activity. The drive to turn a profit might also make pharmacy providers more inclined to offer PrEP screening broadly, and less inclined to turn away clients seeking PrEP due to their personal biases or sexual mores (e.g., related to pre- or extra-marital sex)—both of which are known barriers to PrEP delivery in clinic settings [69–71].
  Additionally, studies in the USA and Kenya have found that pharmacies may reach populations that do not often use clinic-based PrEP services. For example, several studies in the USA [72–74] have found that pharmacies have high potential to reach Black MSM—a population disproportionately burdened by HIV/AIDS with a long history of marginalization from the US medical system. These studies also pointed out that, in addition to being more physically accessible, pharmacies offer a disease-neutral, non-stigmatizing environment and often have strong rapport with the surrounding community, making them a trusted healthcare resource. Perhaps for similar reasons, the fully task-shifted model in Kenya [25••] was able to engage a considerable number of unmarried men—a demographic notably underrepresented in public-sector PrEP programs. Moreover, when comparing the sexual behaviors of clients who initiated PrEP in pharmacy-based versus clinic-based studies in Kenya, we saw notable differences. Specifically, the percent of clients who reported sex with partners of unknown HIV status and multiple concurrent sex partners was considerably higher at pharmacies, while reports of sex with partners known to be living with HIV was considerably lower (Table 4).
  Further evidence that clients who are inclined to initiate PrEP at pharmacies may be disinterested in clinic-based PrEP services and vice versa comes from Kenya. The pilot of the partially task-shifted model for PrEP refills found that, among 41 clients who initiated PrEP at a clinic and refilled their prescription at least once, very few (*n* = 3) opted to refill at a pharmacy [22••]. Moreover, in the pilot of the fully task-shifted model, PrEP clients at their last study visit were referred to nearby public health facilities where they could continue getting PrEP services for free; however, a follow-up survey with 492 clients 3 months following study completion found that 59% (291/492) had stopped using PrEP and, of these, 60% (175/291) said they did so because they did not want to get PrEP from a clinic [75•].
3. **When PrEP is made available in pharmacies, uptake and continuation often matches or exceeds that commonly seen in clinics**. In Kenya, the private pharmacies that participated in pilot studies achieved levels of PrEP initiation and continuation comparable to those of clinics offering PrEP as part of routine service delivery (Table 4). The comparability of these utilization metrics is compelling because it suggests that pharmacies can “perform” just as well as clinics but among a client population that clinics (especially those in the public sector) are less likely to capture.
4. **Most clients and providers who obtain or deliver PrEP services via pharmacies find this delivery setting acceptable; additional evidence on other implementation outcomes (e.g., feasibility, cost) is needed.** In survey and interview studies, pharmacy-based PrEP delivery has generally been perceived by clients as convenient and private and by providers as not overly burdensome to deliver [23•, 25••, 62••]. However, a given model’s acceptability will likely vary based on a number of factors, especially how much clients are charged and the amount of time pharmacies have (or make) available to serve PrEP clients, with the former likely influenced by how profitable PrEP delivery is to pharmacies [23•, 76].
  To date, there is little published evidence on other implementation outcomes, such as feasibility, appropriateness, and cost. One exception is the Kenya-based pilot of the stand-alone model in which clients paid ~ $3 USD to initiate and/or continue PrEP, which found that most clients perceived the model to be highly appropriate and that the financial cost (i.e., cost of implementing the model, including the cost of pharmacy provider time but excluding the cost of the PrEP drugs and HIV test kits donated by the MOH) was $1.52 USD per PrEP initiation visit and $1.38 USD per PrEP continuation visit [77]. Additional research is needed to assess the implementation of pharmacy-based PrEP delivery models, identify context-specific factors that help or hinder implementation, and test strategies to enhance model implementation and sustainability. Such information is critical for informing whether it is worth pursuing pharmacy-based PrEP delivery in a given setting and, if so, how the model might be implemented to circumvent or mitigate potential barriers.
5. **With proper training and oversight, pharmacy providers are capable of safely initiating and managing clients on PrEP and maintaining their privacy**. Despite concerns raised by skeptics, the available evidence on pharmacy-based PrEP delivery indicates that trained pharmacy providers can educate clients about HIV and PrEP, assess clients’ HIV risk, rule out medical conditions that may contraindicate safety, and conduct HIV testing and counseling while maintaining high levels of privacy [78••]. The published evidence also shows that pharmacy providers utilize remote clinicians appropriately, reaching out for consultations and referrals, when appropriate.

**Table 3:** Published findings about select pharmacy-based PrEP delivery models in the USA, South Africa, and Kenya

		Population	Reporting period	PrEP uptake^a^	PrEP continuation^b^	Implementation outcomes^c^	Willingness to pay
**Co-located model** (i.e., no task shifting)							
**Kenya**	Pintye et al. (2023)^21^	Confirmed HIV-negative AGYW (≥ 15–24 years)	Oct. 2020–Mar. 2021 (5 months)	85% (200/235)	*Not assessed*	Acceptability – Clients: High (assessed via interviews) [79•]	After 1 month: 69% (107/155) Median (IQR) amount: 150 (100–200) KES (~ $1 [$0.70–$1.40] USD) per follow-up visit
**Partially task-shifted model**^**d**^							
**South Africa**	Shipp et al. (2023) [23•]	General population age ≥ 18	Aug. 2019–Dec. 2020 (15 months)	*Not reported*	*Not yet published*	Acceptability – Clients: High (assessed via interviews)	*Not yet published*^e^
**Kenya**	Mogere et al. (2023) [22••]	Adults (≥ 18) initiated on PrEP at a clinic	Nov. 2020–Oct. 2021 (11 months)	*Not applicable*	At 1 month: 39% (41/106), with only 1% (3/106) opting to refill at a pharmacy	Acceptability – Clients: Low (assessed via interviews) [79•]	*Not yet published*^e^
**Fully task-shifted model** (i.e., standalone model)							
**USA**	Tung et al. (2018) [68••]	General population age ≥ 18	Mar. 2015–Feb. 2018 (35 months)	97% (695/714)	Still “active in the service” at end of study period:^f^ 54% (372/695)	*None assessed*	*Not assessed*
**Kenya**	Ortblad et al. (2023) [25••]	General population age ≥ 18	Nov. 2020–Oct. 2021 (11 months)	60% (287/476)	At 1 month: 53% (153/287); At 4 months: 36% (103/287); At 7 months: 21% (51/242)	Acceptability – Clients & providers: High (assessed via survey) [80] Appropriateness – Clients: High (assessed via survey)^h^ Cost: Financial cost per initiation visit: $1.52 USD; per continuation visit: $1.38 USD [77] Fidelity: High (assessed via standardized client actors) [78••] Feasibility: *Not yet published*^*e*^	After 1 month: 98% (150/153) Median (IQR) amount: 300 (200–375) KES (~ $2.71 [$1.81-$3.39] USD) per follow-up visit
	Roche et al. (2023) [62••]	General population age ≥ 18	Jan.–Jul. 2022 (6 months)	97% (684/704)	At 1 month: 71% (484/684)		At enrollment: 83% (575/684) Median (IQR) amount: $3.30 ($1.60–4.10 USD) per follow-up visit [77]

*AGYW* adolescent girls and young women, *IQR* interquartile range, *PrEP* pre-exposure prophylaxis, *KES* Kenyan shillings, *USD* US dollars

^a^Among those determined to be eligible to receive PrEP. Example conditions that would render a client ineligible to continue PrEP at a pharmacy include testing positive for HIV and severe PrEP side effects

^b^Denominators exclude clients who did not return for follow-up and those deemed ineligible to continue PrEP at the pharmacy (e.g., due to severe PrEP side effects requiring referral or testing HIV-positive)

^c^As defined in Proctor et al.’s taxonomy of implementation outcomes (Proctor et al. Outcomes for Implementation Research: Conceptual Distinctions, Measurement Challenges, and Research Agenda. Adm Policy Ment Health. 2011; 38(2): 65–76. doi:https://doi.org/10.1007/s10488-010-0319-7). Since client uptake is indicated in the “PrEP uptake” and “PrEP continuation” columns, “adoption” in this column refers to uptake of PrEP delivery by the target provider

^d^Exact task(s) shifted to pharmacy providers varies by model

^e^Not reported in these abstracts but will be reported on in main outcomes paper, currently under review

^f^135 clients were lost to follow-up. Remaining 188 clients transferred to a primary care physician (*n* = 135), relocated out of the area (*n* = 34), and/or decided to stop PrEP due to change in perceived HIV risk (*n* = 40)

^g^Assessed via 5-point Likert-type statements assessing dimensions of acceptability (e.g., burden, affective attitude) from the Theoretical Framework of Acceptability

^h^Assessed using a modified version of the Intervention Appropriateness Measure (Weiner et al. Psychometric assessment of three newly developed implementation outcome measures. Implement Sci. 2017;12(1):108. doi: https://doi.org/10.1186/s13012-017–0635-3)

**Table 4:** Risk behaviors of clients initiating PrEP at different service locations in Kenya

		Pharmacy-delivered PrEP services		Clinic-delivered PrEP services		
		Fully task-shifted model (*N* = 287)	Co-located model (*N* = 235)	Partners Scale-Up; at HIV clinics (*N* = 4898)	PrIYA Program; at MCH clinics (*N* = 2030)	PrIYA Program; at FP clinics (*N* = 278)
**Study description and demographics**	Study duration	11 months (Nov. 2020–Oct. 2021)	5 months (Oct. 2020–Mar.2021)	30 months (Jan. 2017–Jun. 2019)	7 months (Nov. 2017–Jun. 2018)	7 months (Nov. 2017–Jun. 2018)
	Population of interest	*Anyone at risk of HIV infection (*≥ *18 years)*	*AGYW seeking contraception services at private pharmacies (*≥ *15 to 24 years)*	*Anyone at risk of HIV infection (*≥ *18 years)*	*Women attending antenatal or postnatal care (*≥ *15 years)*	*Women of reproductive age (15 to 45 years)*
	PrEP delivery location	*Private community pharmacies* (*n* = 5)	*Private community pharmacies* (*n* = 3)	*Public HIV care clinics* (*n* = 25)	*Public, faith-based, & private maternal & child health clinics* (*n* = 16)	*Public family planning clinics* (*n* = 13)
	Implementation strategy	*Pharmacy provider-led delivery*	*PrEP-dedicated nurse-led delivery*	*Healthcare provider-led delivery*^1^	*PrEP-dedicated nurse-led delivery*	*PrEP-dedicated nurse-led delivery*
	Men	*163/287 (57%)*	*-*	*2257/4898 (46%)*	*-*	*-*
	< 25 years	*127/287 (44%)*	*235/235 (100%)*	*969/4898 (20%)*	*999/2030 (49%)*	*87/278 (31%)*
	Unmarried	*178/287 (62%)*	*159/200 (80%)*	*432/4898 (9%)*	*399/2030 (19%)*	*213/278 (77%)*
**Behaviors associated with HIV risk, past 6 months**	***Men initiating PrEP services***					
	Partner(s) HIV status unknown	136/163 (83%)	-	236/2257 (11%)	-	-
	Partner(s) living with HIV	4/163 (2%)	-	1993/2257 (88%)	-	-
	Inconsistent or no condom use	111/163 (68%)	-	921/2257 (41%)	-	-
	Multiple sexual partners	118/163 (72%)	-	260/2257 (12%)	-	-
	Recurrent sex with alcohol	63/163 (39%)	-	67/2257 (3%)	-	-
	Transactional sex^2^	21/163 (13%)	-	23/2257 (1%)	-	-
	***Women initiating PrEP services***					
	Partner(s) HIV status unknown	96/124 (77%)	-	553/2640 (21%)	1178/2030 (58%)	151/278 (54%)
	Partner(s) living with HIV	7/124 (6%)	-	2098/2640 (80%)	153/2030 (8%)	62/278 (22%)
	Inconsistent or no condom use	92/124 (74%)	-	1150/2640 (44%)	1946/2030 (96%)	31/278 (11%)
	Multiple sexual partners	46/124 (37%)	-	305/2640 (12%)	-	18/278 (7%)
	Recurrent sex with alcohol	27/124 (22%)	-	44/2640 (2%)	-	4/278 (1%)
	Transactional sex^2^	9/124 (7%)	-	44/2640 (2%)	17/2030 (1%)	8/278 (3%)
	***Adolescent girls and young women (15–24 years) initiating PrEP services***					
	Partner(s) HIV status unknown	51/64 (80%)	116/193 (60%)	178/673 (26%)	648/1129 (57%)	64/106 (60%)
	Partner(s) living with HIV	2/64 (3%)	1/200 (< 1%)	478/673 (71%)	48/1129 (4%)	0/106 (0%)
	Inconsistent or no condom use	48/64 (75%)	199/200 (99.5%)	341/673 (51%)	1081/1129 (96%)	11/106 (10%)
	Multiple sexual partners	16/64 (25%)	72/200 (36%)	113/673 (17%)	-	6/106 (6%)
	Recurrent sex with alcohol	8/64 (13%)	30/200 (15%)	7/673 (1%)	-	1/106 (< 1%)
	Transactional sex^2^	7/64 (11%)	53/196 (27%)	21/673 (3%)	8/1129 (< 1%)	2/106 (2%)
**Client outcomes**	***PrEP initiation and continuation at different service locations in Kenya***^3^					
	PrEP initiation (/eligible participants)	287/575^4^ (60%)	200/235 (85%)	4898/NR (N/A)	2030/9376 (22%)	278/1271 (22%)
	PrEP continuation (1 month)	153/287 (53%)	105/200^5^ (53%)	2806/4898 (57%)	786/2030 (39%)	114/278^6^ (41%)
	PrEP continuation (3 months)	106/287 (36%)	-	2135/4898 (44%)	441/2030 (22%)	68/278 (25%)
	PrEP continuation (6 months)	51/287 (21%)	-	1661/4898 (34%)	189/2030 (12%)	30/278 (11%)

*PrEP* pre-exposure prophylaxis, *PrIYA* PrEP implementation in young women and adolescents, *AGYW* adolescent girls and young women, *NR* not reported, *NA* not applicable

^1^Health care providers include nurses, clinical officers, or HIV counselor

^2^Includes participants who bought or sold sex in exchange for money or goods

^3^In accordance with the reports of the implementation studies, we defined PrEP initiation as “documentation of having received a PrEP prescription in clinic records” and PrEP continuation as the “proportion of people initiating PrEP who had a documented PrEP refill within the visit window”.[4]

^4^Among those identified as eligible for PrEP services at the pharmacy (*N* = 476)

^5^Participants that planned to continue PrEP at 1 month and were referred to the nearest PrEP-dispensing health clinic

^6^Returned to collect at least one PrEP refill within 45 days post-initiation

## Potential Pitfalls and Future Directions

With only 6 years to reach the Sustainable Development Goal of zero new HIV infections by 2030 [81], health systems must creatively leverage both their authority and available resources to ensure that HIV prevention tools are available across a multitude of service delivery platforms. Whether it makes sense for a given country to involve private pharmacies in PrEP delivery—and, if so, which models to use—will be highly context-specific and depend not only on the state of the private pharmacy sector (e.g., how robust, organized, and utilized it is), but also on the extent to which the country can address key needs related to governance, financing, and regulation; needs that if unaddressed, can severely delay or blunt the effectiveness of pharmacy-based PrEP delivery.

At the same time, since pharmacy-based PrEP delivery has yet to be widely implemented anywhere and some of the challenges to implementing it (e.g., provider time constraints) are likely to be universal, there is a prime opportunity to develop learning collaboratives whereby implementers from both high- and low-and-middle-income countries exchange experiences, insights, and innovations. Below, we offer general recommendations and research directions (summarized in Table 5), with the understanding that the path to pharmacy-based PrEP delivery will look different in each context.

**Table 5:** Research gaps related to the governance, financing, and regulation of pharmacy-based PrEP delivery

Topic	Subtopic	Possible research directions
Governance	*Professional associations*	Assessing support and identifying potential implementation barriers and solutions to delivering PrEP via pharmacies
	*Delivery model(s)*	Developing and testing models/model modifications and generating data on the reach, utilization, acceptability, feasibility, and cost of different delivery models
Financing	*Public financing*	Conducting costing studies to understand the economic viability of different types of government support for private pharmacy-based PrEP delivery (e.g., government-supplied commodities and personnel)
		Developing and testing ways for private pharmacy providers to be incorporated into national health insurance schemes as PrEP providers
	*Private financing*	Conducting a landscape assessment of current or potential private insurer coverage of PrEP services delivered via private pharmacies (e.g., which PrEP modalities are/would be covered, for whom; client out-of-pocket expenses)
	*Donor financing*	Assessing donor willingness to allow donor-supplied commodities to be delivered in private pharmacies, including if clients charged a fee for provider’s time; exploring other ways donors might be willing to subsidize PrEP services delivered via private pharmacies (e.g., vouchers for key populations)
	*Billing*	Designing and evaluating mechanisms for private pharmacies to request, track, and report on PrEP services rendered and/or commodities used
		Developing and/or testing billing mechanisms for private pharmacies to receive payment for PrEP services rendered (from governments, insurers, and/or donors)
	*Other*	Assessing willingness to pay among key populations and PrEP-eligible members of the general population and willingness to provide PrEP services among different types of private pharmacies (e.g., independent pharmacies, retail chains)
		Testing strategies to address cost barriers for priority PrEP populations (e.g., sliding scale payment systems)
		Conducting time-and-motion studies and unit-cost analyses to understand workflow burden and costs to pharmacies to deliver PrEP; conducting budget impact analyses to help inform governments about potential benefits of investing in pharmacy-delivered PrEP services
Regulation	*Regulations*	Working with regulatory bodies to think through regulations needed to ensure PrEP is delivered with high fidelity at pharmacies
	*Monitoring*	Defining and validating a set of measures for routine monitoring of pharmacy-delivered PrEP services
		Developing and testing strategies for providing feedback to private pharmacies on their adherence to regulations
		Designing and testing the effect of learning networks on pharmacy providers’ PrEP delivery skills and ability to meet regulations

*PrEP* pre-exposure prophylaxis

### Governance

In many countries, professional associations have strong influence over how healthcare is delivered [82] and, not surprisingly, tend to advocate for policies that further the interests of their specific cadre. In places where HIV services have historically been delivered by physicians and nurses, pushback against involving pharmacy providers (especially if this change could be perceived as a threat to earnings) should be expected and, where possible, preempted. In countries considering or actively pursuing pharmacy-based PrEP delivery, ministry leaders at the national and subnational level should convene professional associations of doctors, nurses, pharmacy providers, and ancillary service providers to understand the landscape of players, their views on potential implementation barriers (especially “deal-breakers”), and possible solutions, including adaptations to the intervention, modifications to the delivery model, and/or implementation strategies that may address the issues raised. Where appropriate, governments should also involve professional associations in the development and evaluation of curricula for pharmacy provider pre-service and in-service training so that concerns about pharmacy provider competency to deliver PrEP can be addressed proactively. Lastly, there needs to be a mechanism through which pharmacy providers delivering PrEP can access clinical support from HIV specialists.

Research that can help countries address these governance needs include stakeholder and intervention mapping exercises, surveys and interviews with representatives of professional associations, and studies testing model adaptations and/or implementation strategies to address concerns raised.

### Financing

In recent years, global health authorities and donor agencies, including the WHO [83], PEPFAR [84], and USAID [85], have expressed a growing interest in engaging the private sector in HIV service delivery; however, there is currently no global guidance around HIV service delivery via private pharmacies nor large-scale investments specific to this delivery venue. Getting pharmacy-based PrEP delivery to occur at scale—and at a price affordable to a meaningful portion of the target demographic—will likely require not only alignment of country, donor, and private sector priorities but also innovative financial strategies at multiple levels (e.g., at the client level, vouchers; at the clinic level, sliding scale payment schemes; at the national level, policies requiring national and private health insurers to cover PrEP services).

If a country seeks to scale up pharmacy-based PrEP delivery quickly, it should commit to financing, at a minimum, the cost of PrEP drugs at pharmacies, even if clients pay a service fee. In places where PrEP drugs are largely donor-supplied and donors (e.g., PEPFAR) have historically prohibited charging clients service fees, countries should advocate for this change. Bi- and multi-lateral donor agencies (e.g., Global Fund, PEPFAR) should also consider establishing commitments specifically for innovations related to pharmacy-based PrEP delivery—such as incorporating private pharmacies into government supply chains—and use their influence to facilitate enabling legislation or agreements, such as strategic purchasing partnerships between governments and private sector entities. In settings like the USA where most PrEP drugs are procured directly from suppliers, governments should use their power and influence to get private insurers to cover PrEP services (or enforce existing mandates) while also adequately funding state-sponsored PrEP assistance programs for uninsured clients.

Importantly, if any portion of pharmacy-delivered PrEP services is to be covered by a third-party payer—such as government, donor, or private insurer—then a mechanism for pharmacy providers to document and bill for their services must be established. Catalytic donor funding could support this by financing pharmacy integration into government health management information systems (HMIS) and/or the refinement of technology already in use at private pharmacies (e.g., point-of-sales systems) to allow relevant information to be fed directly into HMISs. Financial strategies should also be solicited directly from the private sector, especially social entrepreneurs, venture capitalists, and private philanthropic organizations with relevant expertise.

Lastly, when deciding which model(s) to pursue, countries should carefully consider human resource requirements. Both the partially and fully task-shifted models enable private pharmacies to deliver PrEP without significant additional human resources—an important advantage for countries that face chronically low workforce density, provided that pharmacy providers can be appropriately compensated for their time [83, 84].

Research that could inform decision-making related to financing includes assessments of willingness to pay among PrEP priority populations and willingness to provide among pharmacy providers, estimates of price elasticity (how supply and demand for PrEP change when the price of PrEP services change), cost effectiveness assessments, and time-and-motion studies and unit-cost analyses to understand the workflow burden and costs to pharmacies to deliver PrEP.

### Regulation

Lastly, pharmacy-based PrEP delivery cannot go to scale without a clear plan for accreditation and regulation. Governments will need to not only establish standards that private pharmacies must meet to deliver PrEP but also set regulations and determine how compliance will be monitored and enforced.

Research directions include creating and validating a simple set of measures to include in regulations, such as measures for accrediting pharmacy providers who complete training and for assessing quality, including fidelity to the core components of PrEP delivery (e.g., HIV testing); assessing new technologies for providing feedback to private pharmacies on their adherence to regulations (e.g., artificial intelligence technology to verify that HIV testing is done consistently and accurately prior to prescribing [86]); and designing and testing the effect of in-person or virtual learning networks on pharmacy providers’ ability to crowdsource knowledge (e.g., ways to efficiently meet regulatory requirements) and improve their PrEP delivery skills.

## Conclusion

Early evidence from several countries provides proof of concept that private pharmacies can be leveraged in a variety of ways for PrEP delivery and that doing so may increase PrEP coverage and utilization, especially among populations not reached by clinic-based services; however, additional research and programmatic evidence is needed to understand how best to implement and finance these models to maximize impact. Importantly, pharmacy-based PrEP delivery is still nascent everywhere, thus presenting an opportunity for shared learning and innovation among high and low-and-middle income countries. Countries with robust private pharmacy sectors and high HIV burden should focus on aligning key areas related to governance, financing, and regulation that have proven critical to pharmacy-based PrEP delivery while, at the same time, pursuing an ambitious research agenda to generate information for decision-making. Without this parallel track, we may miss our opportunity to meet global HIV prevention goals and prevent thousands of unnecessary HIV infections.
